# 

**Published:** 1911-02-15

**Authors:** 


### ANNOUNCEMENTS.

---

THE CROUSE-TAGGART DEAL. Referring to a recent circular letter to the profession entitled "Dental Protective Association Agreement with Dr. Taggart," which letter requests members of the dental profession to "read the accompanying *Amended* By-Laws," and "immediately sign and return the same together with your check," and to pay Taggart "before the entry of any decree or judgment finding Taggart's patent valid," I, as chairman of the committee conducting the defense for the profession in the Taggart test suit, respectfully ask your attention from another viewpoint.

The Dental Protective Association collected large sums of money under the alleged chief object of defending "any of its members in any of the States or Territories when prosecuted for the infringement of patents the validity of which has not been fully established.

The conceiving and entering into an agreement with Dr. Taggart, by which the Dental Protective Association shares with him the pecuniary benefits to be derived from the third party.—the dental profession being made the third party without its knowledge or consent is to so change and pervert the stated object of the D. P. Association that it might appear to have authority to use its funds, its influence, and its machinery in affiliation with the patentee for the collection of license fees for the use of the Taggart process patent. This being accomplished, the next move is the sending out of this circular letter which expressly states the object of making collections of license fees "*before the entry of any decree or judgment,*" *etc.*

You should now remember the fact that about three years ago Dr. Taggart entered suit under circumstances that then appeared to be a sincere attempt to test the validity of his patent, and the profession was forced, by reason of his exorbitant demands and extraordinary claims, to join with him in the test. But Dr. Taggart has delayed the progress of the suit while overtures were being made from one to another of his friends, and finally negotiations were concluded with his neighbor, Dr. Crouse of the D. P. Association. This unseemly and undue delay has impelled the court to issue an order in behalf of the defense which compels the plaintiff to close his case next month (February). Therefore, notwithstanding this extraordinary interpolation and unseemly interference with a pending suit, the defense in behalf of the profession will be continued to the only legitimate end, a final decision, and without the least taint of compact or alliance with any interest.

Neither the Committee nor any of the many members of the profession supporting the defense have the slightest personal prejudice nor the least objection to any payment to Dr. Taggart that may be made by any one of those who Dr. Crouse, at this late day, comes out and says, "felt they were in some way indebted to Dr. Taggart." But there is objection to Dr. Crouse, acting in behalf of the Dental Protective Association and of Dr. Taggart, interfering with a pending suit, and representing to the profession that the validity of the patent will probably be sustained in connection with an implied threat and a direct request for the immediate signing and returning of the *amended* by-laws, together with check for the payment of Dr. Taggart "before the entry of any decree or judgment" in this case.

Dr. Taggart did not seek, so far as we know, an amicable and equitable settlement with the defendant profession. He selected the nominal defendant and the jurisdiction best suited to his purpose and forced the beginning of a suit that called for a defense in behalf of the profession in general.

Scores and hundreds have joined the committee in the difficult task of raising funds to meet the necessarily heavy expenses of taking testimony in various States, but the interest of the profession has not and shall not suffer because of the difficulty of raising much needed funds. The defense is and all the time has been anxious to reach a conclusion. The plaintiff delays while negotiations are carried on for the purpose of giving Dr. Crouse control of more money that may be used to prevent the testing of the validity of patents which are granted by the Patent Office without a contest in the routine procedure of that office. The condition of the profession would be deplorable if there were no opportunity for testing the validity of patents granted after a hearing of only the patentee. What the condition will be if our associations, organized and supported for the sole purpose of testing the validity of patents, pervert their objects and seek to involve the profession and embarrass its defenders in pending patent suits, we leave to you to judge. But of this be assured, the recent order of the Court compelling Taggart to close his case next month, will give us the opportunity we need to force the case to an early adjudication. We have the utmost confidence in the judgment of our lawyers who assure us that the defense is more than abundantly sufficient to win a verdict for the profession and to save it from the yoke of a process patent and an office license of the Goodyear-Patent kind. Send Crouse and Taggart "immediately" all the checks you wish before the suit is decided by the Court, but don't do so with the idea that the defense conducted in your behalf lacks in evidence, in common sense or in legal talent. The defense committee lacks nothing but money with which to force the plaintiff to submit the insufficient evidence on which his case rests, but in some proper way will meet the expense.

Do a little thinking for yourself on Crouse's propositions.

If the validity of the Taggart patent will probably be sustained, why does the plaintiff have to be forced by the defense, after about three years, to submit his case? And, why has the plaintiff come down from the exorbitant demands to the meager sum for which he negotiates through his neighbor, Dr. Crouse? Would not the value of the patent and license to use the method increase with the near approach of a verdict sustaining the claims of the patentee? And again, why did our great defender against patents "the validity of which had not been fully established" go off for years on a Rip Van Winkle sleep while the profession has been strenuous in taking evidence in various States on which the Court will reach a decision Dr. Crouse now seeks to forestall? • And, further, why should Dr. Crouse seek and claim to have legally secured a far reaching perversive change from the objects and purposes for which the fees we have paid the Protective Association may beus ed?

The professions' defenders in this case are not seeking a compromise nor affiliation with any patentee or organization but are using every dollar the committee can raise to speed the day when this case will be decided on its merits as shown by the evidence in the case, and be assured no fear need be felt because of the evidence so far recorded.

Fraternally,

MARK F. FINLEY,

*Chairman, Committee for the Defense.*

Washington, D. C., January 6th, 1911.

---

NEW ORLEANS ODONTOLOGICAL SOCIETY. At the annual meeting of the Odontological Society of New Orleans held January 11th, 1911, the following officers were elected to serve for the ensuing year: E. L. Fortier, D.D.S., President; St. Clair Duke, D.D.S., Vice-President; Alfred A Leefe, D.D.S., Secretary-Treasurer; Executive Commitee, F. H. Field, D.D.S.; Chairman; T. J. Wingrave, D.D.S. E. B. Ducasse, D.D.S.

MICHIGAN STATE DENTAL SOCIETY. The Michigan State Dental Society will meet the week beginning April 10th, 11th and 12th, at Grand Rapids. This is an earlier date than usual and should be kept in mind.

---

RESOLUTIONS PASSED BY THE NATIONAL DENTAL ASSOCIATION. *Resolved*, That it is the conviction of the National Dental Association that the metric system shall become the accepted standard of weights and measures in all professional and commercial transactions.

To accomplish this purpose at as early a date as may be practicable, it is urged that all physicians, dentists, druggists and dealers in precious metals be urged to put into practice this system of weights and measures at once. It is further

*Resolved*, That this resolution be published with the proceedings of the Society, and a copy sent to all the dental journals and to the Secretary of the U. S. Pharmaceutical Convention, Dr. Murray Galt Motter, 1841 Summit Place, Washington, D. C.

---

ST. LOUIS DENTAL COLLEGE. The annual clinic of the Alumni Association of the St. Louis Dental College will be held in the College Building on Saturday April, 15, 1911. All ethical practitioners are cordially invited to attend.

FRANCIS P. MAHON, *Sec'y.*.

---

SOUTHERN BRANCH NATIONAL DENTAL ASSOCIATION. The Southern Branch of the National Dental Association will meet in Atlanta, Ga., Tuesday, April 4th, 5th and 6th, at 10 A. M. Headquarters will be at the Piedmont Hotel. All business sessions will be held there and the Manufacturers Exhibit also.

The Railroads have given the following rates: A fare and one-half plus 50 cents in all territory south of the Mason and Dixon lines, and east of the Mississippi, provided there are two hundred attendants who travel by rail. Those living outside of this district, will please communicate with the Corresponding Secretary, Dr. W. G. Mason, Tampa, Fla., as to rates attainable.

The various committees are hard at work, and the meeting promises to be an exceptionally good one. The time selected for the meeting is ideal in this section, and as it is well in advance of the commencement exercises—it is expected that we will have an unusually large attendance.

A more complete announcement will be made in next months journal, but it is well to make your other arrangements fit in with these dates. C. M. BARNWELL,

*for Committee on Arrangements.*

---

ILLINOIS STATE DENTAL SOCIETY. The forty-seventh annual meeting of the Illinois State Dental Society will be held at Peoria, May 9, 10, 11, 12, 1911.

J. P. LUTHRINGER, *Chairman*, Local Arrangem. Com., Peoria.

J. F. F. WALTZ, *Sec'y*, Decatur.

---

CHICAGO DENTAL SOCIETY. At the regular meeting of the Chicago Odontographic Society held Dec. 20th, 1910, in the Chicago Public Library Building, the name of the society was changed from that of the Chicago Odontographic Society to the Chicago Dental Society and the following officers elected for the ensuing year: President, G. W. Dittmar; Vice-President, G. N. West; Treasurer, F. E. Roach; Secretary, T. L. Grissmore; Librarian, E. D. Coolidge Board of Directors, elected: R. Beck, L. F. Bryant, L. E. Bake, J. P. Smith; Board of Directors now stands: R. Beck, G. M. Gallie, 1 year; A. D. Black, L. F. Bryant, 2 years; J. P. Smith, L. E. Bake, 3 years; Board of Censors, P. B. D. Idler, Chairman; H. C. Piesch, A. M. Hewett; Organization Committee, W. H. G. Logan, Chairman, F. W. Gethro, R. J. Cruise.

